# Reliability of LoSCAT score for activity and tissue damage assessment in a large cohort of patients with Juvenile Localized Scleroderma

**DOI:** 10.1186/s12969-018-0254-9

**Published:** 2018-06-18

**Authors:** Anna Agazzi, Gloria Fadanelli, Fabio Vittadello, Francesco Zulian, Giorgia Martini

**Affiliations:** 1 Paediatric Rheumatology Unit, Department of Woman and Child Health, University of Padova, Via Giustiniani 2, 35128 Padova, Italy; 2 Paediatric Unit, Santa Chiara Hospital, Trento, Italy

**Keywords:** Localized scleroderma, Outcome measures, LoSCAT, Thermography, Morphea, Children

## Abstract

**Objectives:**

To assess reliability of the two indexes of Localized Scleroderma Cutaneous Assessment Tool (LoSCAT), the modified Localized Scleroderma Skin Severity Index (mLoSSI) and the Localized Scleroderma Skin Damage Index (LoSDI), when applied by clinicians with different experience in scoring and managing patients with JLS. Secondary aim was to compare LoSCAT and infrared thermography (IRT) in monitoring lesions over time.

**Methods:**

Consecutive children with Juvenile Localized Scleroderma (JLS) were blindly evaluated by three examiners with different experience in Paediatric Rheumatology and with no experience in LoSCAT use. At each visit, patients were assessed by LoSCAT and IRT. Sensitivity to change of LoSCAT and IRT was assessed in a group of patients 3–6 months later. Inter-rater reliability was assessed by Intraclass Correlation Coefficient (ICC) and variance analysis (ANOVA).

**Findings:**

Forty-seven patients (129 lesions) entered the study, and 26 (79 lesions) were re-evaluated with same modality after 4.5 (SD 1.5) months. mLoSSI showed excellent inter-rater reliability expressed by ICC 0.965 confirmed by ANOVA. Similarly, inter-rater reliability for LoSDI was good (ICC = 0.774) but worse concordance among examiners was observed. A comparable improvement of mLoSSI in all anatomic sites was noted by all examiners in 79 lesions examined in two subsequent visits and was consistent with thermography.

**Conclusions:**

Different clinical experience in JLS did not influence clinical judgement in mLoSSI which showed excellent concordance, whereas LoSDI is less precise in damage assessment and not completely reliable in monitoring skin changes. Infrared thermography confirms to be a helpful tool for detecting disease activity and reliable in monitoring lesions over time.

Juvenile Localized Scleroderma (JLS) is a characterized by an initial phase of inflammation followed by skin fibrosis due to collagen deposition, sclerosis and dermal atrophy [1]. Although not a lethal disease, JLS can cause deformities like subcutaneous fat loss, joint contractures, growth discrepancies and aesthetic damage resulting in psychological consequences [2, 3].

Assessment and monitoring of inflammation and tissue damage is crucial in JLS, but the lack of standardized and reliable outcome measures represented a limitation for clinicians over the years. Several assessment tools such as Computerized skin score (CSS) [4], infrared thermography (IRT) [5], laser doppler flowmeter [6], doppler ultrasound [7–9], magnetic resonance imaging (MRI) [10, 11] and, more recently, Cone Beam Computed Tomography (CBCT) have been proposed [12]. The need for an easy to use, fast in daily clinical practice and inexpensive outcome measure led to development of the Localized Scleroderma Cutaneous Assessment Tool (LoSCAT) [13, 14]. It is composed of two indexes: the modified Localized Scleroderma Skin Severity Index (mLoSSI) and the Localized Scleroderma Skin Damage Index (LoSDI).

Our aim was to evaluate whether LoSCAT scores correlate well among physicians with different degrees of experience in scoring and managing patients with JLS, and secondarily to compare LoSCAT and IRT in monitoring the lesions over time.

## Patients and methods

A longitudinal observational study of patients with JLS was conducted: consecutive patients diagnosed according to the 2006 Padua Classification criteria [1] were evaluated by three examiners with different degrees of experience in JLS management: a senior paediatric rheumatologist (examiner 1), a paediatric rheumatology fellow (examiner 2) and a medical student (examiner 3).

None of the three examiners had experience in LoSCAT scoring and no specific training was performed. In fact, in order to evaluate whether learning the use of this clinical instrument was feasible and simple, each examiner independently studied the definitions of the LoSCAT domains scores as reported in previous studies [13, 14]. They blindly assessed all patients by LoSCAT, considering 18 anatomic sites both for mLoSSI and LoSDI (head, neck, chest, abdomen, upper back, lower back, right and left arms, forearms, hands, thighs, legs and feet). The mLoSSI is composed by three domains of disease activity: new lesion/lesion extension (N/E) with score 0 or 3, erythema (ER) considering the lesion’s edge and skin thickness (ST) with score from 0 to 3. The domains representative of tissue damage forming LoSDI are dermal atrophy (DAT), subcutaneous atrophy (SAT) and dyspigmentation (DP), all scored from 0 to 3 [13, 14].

For each body area the most representative lesion and the worst score for each domain were considered. All lesions were compared with contralateral area or ipsilateral skin areas.

During each visit all patients were examined with same infrared camera (ThermaCAM PM695, FLIR systems AB, Stockholm, Sweden), at controlled temperature room, after 20 min of acclimatization. Lesions were considered positive when warmer 0.5 °C than surrounding area or contralateral limb.

To evaluate the sensitivity to change of LoSCAT a group of patients were reassessed with same modality during a subsequent routine follow-up visit, 3–6 months later. The relative variations for activity detected by mLoSSI and IRT and for damage evaluated by LoSDI were calculated detracting the value at second visit from the one at first divided by first visit ((v_2_ – v_1_)/v_1_).

Statistical analysis included Intraclass Correlation Coefficient (ICC), Spearman’s Rho coefficient and analysis of variance (ANOVA). Inter-rater reliability was interpreted as follows: ICC values range 0.75–1 excellent reliability, 0.4–0.74 good reliability, < 0.4 poor reliability. All analyses were performed by using IBM SPSS (Vers. 18.0).

## Results

### Patients

Clinical characteristics of patients are summarized in Table 1: 47 subjects with mean age at JLS onset of 7.3 years (SD 4.16) entered the study. Average age at diagnosis was 8.6 (SD 3.75) indicating a mean diagnostic delay of 1.2 years (SD 1.46). Patients were 30 females and 17 males with a mean age of 13.4 years (SD 5.19) and mean disease duration of 6.1 years (SD 4.46, range 0.39–18.40).

**Table 1:** Clinical characteristics of 47 patients included in the study. Data presented as n (%) and age, diagnostic delay and disease duration as mean ± S.D

Gender	Variable	No. 30/17
	F/M	
JLS Subtype		No. (%)
	Linear Scleroderma	30 (63.8)
	limbs/trunk	11(23.4)
	face	19 (40.4)
	PRS	11(23.4)
	ECDS	8 (17)
	Circumscribed Morphea	7 (14.9)
	Generalized Morphea	4 (8.5)
	Mixed	6 (12.8)
Treatment	MTX	21 (44.7)
	MTX, MMF	3 (6.4)
	PDN	1 (2.1)
Age (yrs.)		mean ± S.D.
	At onset	7.34 ± 4.16
	At diagnosis	8.57 ± 3.75
	At v1^a^	13.40 ± 5.19
Diagnostic Delay (yrs.)		1.23 ± 1.46
Disease Duration at v1^a^ (yrs.)		6.06 ± 4.46

^a^*v1* first study visit, *JLS* Juvenile Localized Scleroderma, *PRS* Parry Romberg Syndrome, *ECDS* en coup de sabre, *MTX* Methotrexate, *PDN* Prednisone, *MMF* Mycophenolate Mofetil, *S.D* Standard Deviation

Clinical subtypes were linear scleroderma (63.8%), circumscribed morphea (14.9%), mixed sub-type (12.8%) and generalized morphea (8.5%).

Nineteen patients were not active (defined by absence of new or extended lesions, thermography negative and off therapy for more than 2 years); 6 patients were defined active (presence of at least one new or extended lesion and thermography positive) of which 4 had new onset disease and 2 were flares. The remaining patients were considered stable (no new or extended lesion and ongoing treatment from less than 2 years).

Overall 129 lesions were examined, each patient having mean 2.7 lesions (median 2, range 1–12). At first evaluation, most patients (53.2%) were on systemic treatment: 21 (44.7%) with methotrexate (MTX), 3 (6.4%) with mycophenolate mofetil (MMF) + MTX and 1 with PDN, one was treated with topical tacrolimus. Among the 21 remaining off treatment patients 17 had been treated with systemic agents, 2 with topical (1 tacrolimus, 1 steroids) and 2 had received no treatment.

Twenty-six patients (79 lesions) were re-evaluated after mean 4.5 (SD 1.5) months with 20/26 still on systemic treatment.

### Inter-rater reliability

**Fig. 1:**
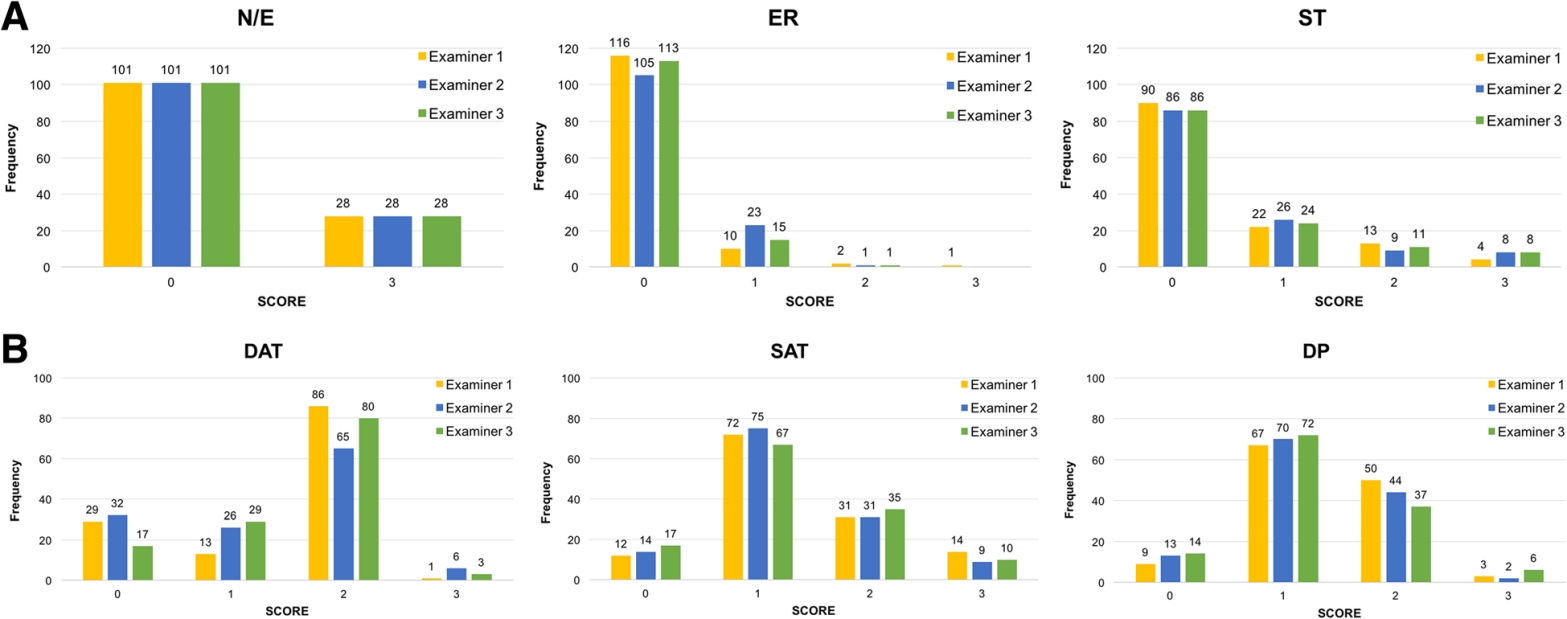
Frequency of different scores for each domain of mLoSSI (**a**) and LoSDI (**b**) at baseline visit. Bars represent the frequencies of each domain score. Distribution recorded by three examiners from 129 lesions. N/E = new lesion/lesion extension, ER = erythema, ST = skin thickness, DAT = dermal atrophy, SAT = subcutaneous atrophy, DP = dyspigmentation

The inter-rater reliability for mLoSSI on 129 lesions was excellent as ICC was 0.965 (95% CI 0.954–0.974), and ANOVA confirmed that mean scores by assessors were similar (F test = 1.740, *p* = 0.178). Concordance in activity domain frequencies was very good, as all 3 physicians evaluated 28 lesions as new or enlarged. Both for ER and ST score 0 was the most frequently attributed by examiners, as shown in Fig. 1a.

Damage domain frequencies were more heterogeneous. The most frequent score for DAT was 2, while for SAT and DP was 1 (Fig. 1b). Indeed, inter-rater reliability was very good for LoSDI (ICC 0.774, CI 95% 0.711–0.827) but ANOVA showed that the mean scores for disease damage were discordant among examiners (F test = 4.524, *p* = 0.012).

The correlation between the three examiners for mLoSSI score as provided by r_s_ in Spearman’s Rho were 0.869, 0.842 and 0.830 between I + II examiner, I + III examiner and II + III examiner, respectively (*p* < 0.0001). For LoSDI correlation was 0.707, 0.788 and 0.782 between I + II examiner, I + III examiner and II + III examiner, respectively (*p* < 0.0001).

### Anatomic sites reliability

Inter-rater reliability of mLoSSI and LoSDI scores according to different sites was evaluated. Paired areas were grouped together so 11 anatomic areas were considered: head, neck, chest, abdomen, back, arms, forearms, hands, thighs, legs and feet. Correlation between examiners showed excellent reliability for mLoSSI in all body sites, with ICC ranging from 0.943 (CI 95% 0.856–0.983) on legs to 0.992 (CI 95% 0.975–0.998) on chest. Overall reliability was good for LoSDI with range of ICC from 0.337 (CI 95% -0.092 – 0.780) on chest to 0.821 (CI 95% 0.394–0.978) on neck.

### Change over time

**Fig. 2:**
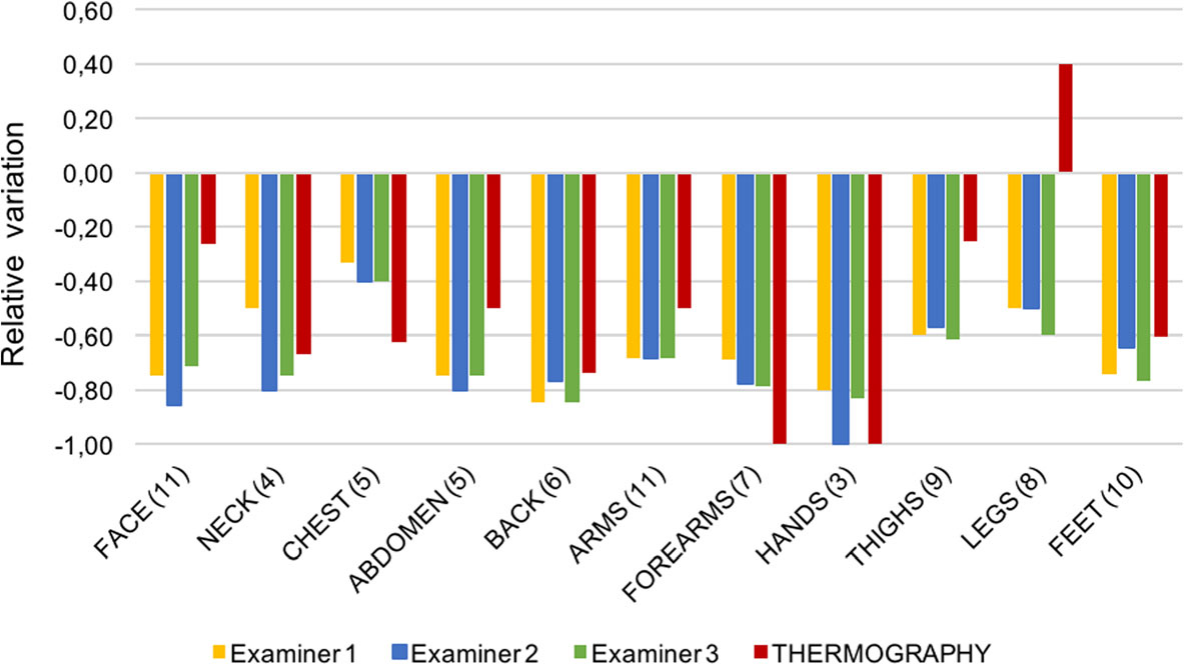
Relative variation between first and second visit for mLoSSI score and IRT. Bars represent the relative variation for the three examiners and thermography in 11 different anatomic areas

Twenty-six patients (79 lesions) were reassessed with same procedure after 4.5 (±1.5) months. A significant reduction of activity by mLoSSI was observed in all body areas by all examiners. This result was consistent with IRT showing a decrease of hyperthermia in all regions except legs. This observation was due to one patient with disease flare in which IRT detected a significant hyperthermia, while mLoSSI score was unchanged (Fig. 2).

**Fig. 3:**
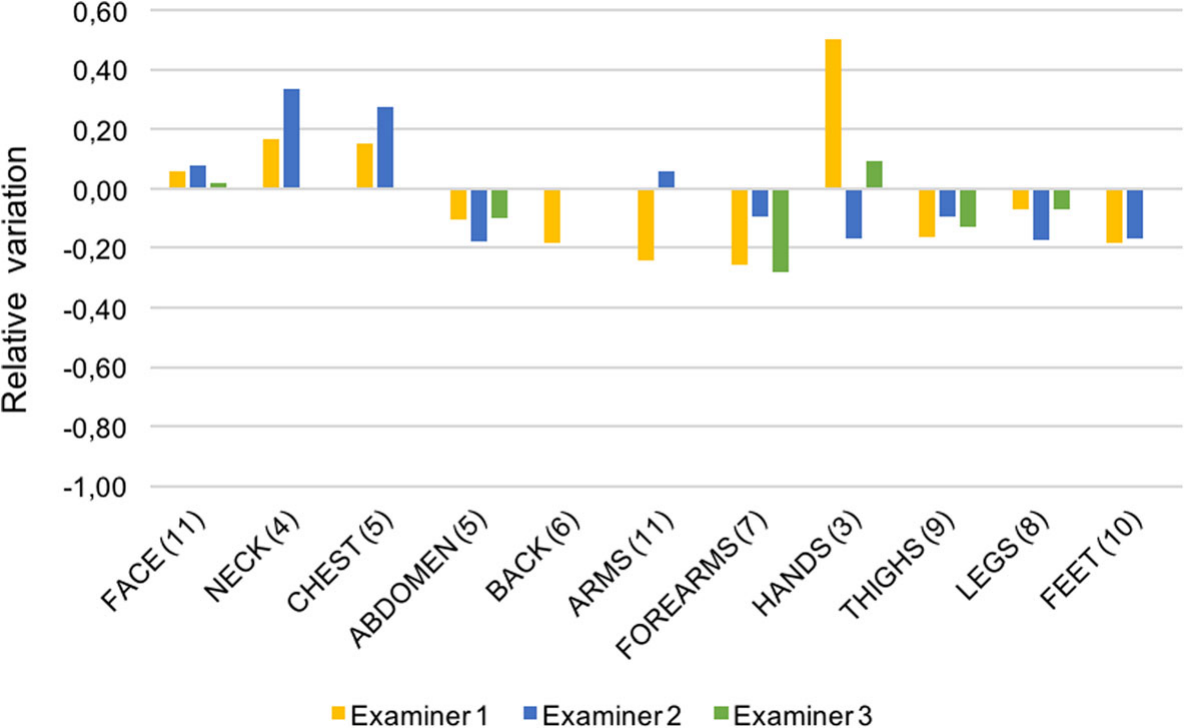
Relative variation between first and second visit for LoSDI score. Bars represent the relative variation for the three examiners in 11 different anatomic areas

The analysis of LoSDI showed more discordant results, although with very small variations mainly ranging from + 0.3 to − 0.3, as illustrated in Fig. 3. Values of damage increased on face, neck, chest and hands, whereas in other sites decreased.

## Discussion

One of the open issues in JLS is assessment of extent of inflammation and tissue damage. The lack of reliable and standardized outcome measures has represented, over the years, a significant limitation for disease clinical monitoring, assessment of therapeutic efficacy and development of therapeutic trials. Indeed, neither clinical examination alone nor lab tests can be considered as reliable indicators of disease activity, like in Juvenile Idiopathic Arthritis (JIA) or Systemic Lupus Erythematosus (SLE).

Aim of present study was to evaluate whether the clinical score LoSCAT can be helpful for clinicians with low experience in scoring and in management of JLS in assessment and monitoring of patients. Furthermore, we compared mLoSSI with IRT, a reliable non-invasive tool with limited availability in pediatric rheumatology centers [5]. Comparison of IRT with LoSDI was not considered since lesions with severe atrophy and fat loss appear falsely positive on IRT [5]. In previous studies, LoSCAT was found to be reliable and sensitive to change and indeed, distinguishing the aspects of activity and tissue damage, allowed physicians to monitor these two conditions separately [15, 16].

One important result of our study is that mLoSSI showed excellent inter-observer agreement (ICC = 0.965) independent from physician’s experience, thus confirming results of previous studies [13]. Indeed, we showed that the site of the lesions did not affect mLoSSI reliability which was very good also in areas like neck, face, hands and feet, difficult to evaluate and demonstrated that no specific training in scoring or in JLS assessment is necessary to use this clinical tool effectively.

In monitoring over time, we observed that mLoSSI was reliable in picking up changes in disease activity, as inter-observer concordance was excellent. Furthermore, mLoSSI variations were consistent with an advanced technology such as IRT therefore confirming that this simple clinical tool can be helpful for monitoring activity in any outpatient setting.

The LoSDI damage evaluation showed more heterogeneous results as ICC was very good (ICC 0.774) but variance analysis indicated low concordance between examiners. In fact, although a similar trend between scores of examiners was observed, they differed in several points. This may be partially explained by the small number of lesions in some body areas and by difficulty in performing clear-cut distinction between degrees of DAT and SAT, probably influenced by clinician’s experience and absence of an appropriate training. The small variations observed in LoSDI scores over an average of 4 months’ time are quite expectable as it evaluates relatively stable cutaneous features.

LoSCAT do not include the evaluation of extracutaneous manifestations, such as bone deformities, joint contractures, central nervous system involvement etc. and has some limitations also in detecting changes in deeper layers. Dermal and subcutaneous fat thickness as well as fascia, muscle and bone involvement can be successfully evaluated by high-frequency Doppler ultrasound, MRI and, on face, by CBCT [9–13]. These tools, combined with clinical assessment, may help in more precise definition and monitoring of tissue damage.

Nevertheless, we demonstrated that LoSCAT, based on simple clinical evaluation, showed good reliability in evaluating active lesions over time, in fact different clinical experience in JLS did not influence clinical judgement in active lesions, while in damage assessment LoSDI was less precise and not completely reliable in monitoring skin changes. IRT confirmed to be a helpful tool for detecting disease activity and reliable in monitoring lesions over time.

## Abbreviations

ANOVA: Analysis of Variance
CSS: Computerized Skin Score
DAT: Dermal atrophy
DP: Depigmentation
ER: Erythema
ICC: Intraclass Correlation Coefficient
IRT: Infrared Thermography
JLS: Juvenile Localized Scleroderma
LoSCAT: Localized Scleroderma Cutaneous Assessment Tool
LoSDI: Localized Scleroderma Skin Damage Index
mLoSSI: Modified Localized Scleroderma Skin Activity Index
MMF: Mycophenolate Mofetil
MTX: Methotrexate
N/E: New/Enlarged lesion
PDN: Prednisone
S.D.: Standard Deviation
SAT: Subcutaneous atrophy
ST: Skin thickness
